# Therapeutic Prospects of Ketamine for Cataplexy in Narcolepsy Type 1: Evidence from a Human Case and a Murine Model

**DOI:** 10.1002/ana.78343

**Published:** 2026-08-28

**Authors:** Francois Ricordeau, Sebastien Arthaud, Leila Langbour, Silvia Melzi, Aurelie Richard‐Mornas, Hannah Doudoux, Benjamin Rolland, Laure Peter‐Derex, Christelle Peyron

**Affiliations:** ^1^ Center for Sleep Medicine Hospices Civils de Lyon, Université Claude Bernard Lyon 1 Lyon France; ^2^ Department of Functional Neurology and Epileptology Hospices Civils de Lyon Lyon France; ^3^ Lyon Neuroscience Research Center, CNRS UMR 5292, INSERM U1028 Université Claude Bernard Lyon 1 Lyon France; ^4^ Medipole de Savoie Challes les Eaux France; ^5^ Service universitaire d'addictologie de Lyon (SUAL), Hospices Civils de Lyon, CH Le Vinatier Bron France

## Abstract

Narcolepsy type 1 is characterized by cataplexy, for which current treatments targeting monoaminergic, histaminergic, and GABAergic pathways may be ineffective or poorly tolerated. We report the case of a 34‐year‐old man with narcolepsy type 1, intolerant to standard therapies, who experienced marked and sustained cataplexy reduction with morning intranasal ketamine, with symptom recurrence upon discontinuation. To confirm this observation, ketamine was compared with vehicle in a crossover study in orexin knockout mice implanted for polysomnography. Ketamine dramatically reduced time spent in cataplexy (−76%, *p* = 0.008) without affecting sleep architecture. These translational findings suggest ketamine as a potential anticataplectic agent, warranting further clinical investigation. ANN NEUROL 2026;100:1023–1027

Narcolepsy type 1 (NT1) is a rare, chronic neurological disorder characterized by sleep‐related symptoms secondary to orexin (hypocretin) deficiency, such as excessive daytime sleepiness, sleep‐related hallucinations and paralysis, disrupted night‐time sleep, and cataplexy.^1^ Current cataplexy treatments target monoaminergic, histaminergic and GABAergic pathways, with recent advances involving orexin‐based approaches. However, some patients experience suboptimal efficacy or poor tolerance, highlighting the need for alternative therapies. Here, we report a robust anticataplectic effect of ketamine, evidenced by a clinical case report of a patient with NT1, and supported by experimental data from a murine model of NT1.

## Methods

### Clinical Case Report

We report the case of a 34‐year‐old man with active smoking and a history of polysubstance use (including LSD, amphetamines and intranasal cocaine). Informed consent was obtained from the patient, and publication of this case report was approved by the local Ethics Committee of the Hospices Civils de Lyon (#26‐240).

In 2016, the patient developed sleep paralysis and hallucinations, as well as typical cataplexy with sudden falls triggered by laughter. Diagnostic evaluation confirmed NT1: the initial Multiple Sleep Latency Test showed a pathological mean sleep onset latency (1.5 min) and 3 sleep‐onset rapid eye movement (REM) periods across 5 naps opportunities; genetic testing was positive for the HLA‐DQB1*06:02 allele; and cerebrospinal fluid analysis confirmed profound orexin‐A deficiency (<50 pg/mL).

Pharmacological treatment proved challenging due to poor efficacy and/or tolerance toward the main wake‐promoting agents (modafinil and methylphenidate were associated with anxiety, pitolisant was considered ineffective) and anticataplectic medications (venlafaxine was associated with mood disturbances and erectile dysfunction, fluoxetine caused gastrointestinal side effects). Given the patient's history of polysubstance use, sodium oxybate was not considered an appropriate therapeutic option. In 2017, the patient initiated recreational ketamine use and reported a marked improvement in cataplexy. By 2019, he had transitioned to ketamine exclusively, taken in the morning, with sustained control of REM sleep‐related symptoms, including cataplexy, sleep paralysis, and hallucinations. Ketamine was prepared from crystals that the patient ground into a powder and administered intranasally. He was unable to provide a reliable estimate of the dose used. No dissociative or psychotomimetic effects were reported. The anticataplectic effect appeared directly tied to ketamine use: cessation led to symptom recurrence after approximately 10 days, with immediate resolution upon resumption. Over time, the patient noted a diminished duration of effect, with cataplexy returning within a few hours to 2 days post‐dose depending on the formulation used.

Notably, although excessive daytime sleepiness was initially managed through strategic napping (Epworth Sleepiness Scale score = 2/24 in 2016), this symptom significantly worsened over the years (Epworth Sleepiness Scale = 19/24; Narcolepsy Severity Scale = 35/57 in 2023).

To test whether this clinical observation of an anticataleptic effect of ketamine could be replicated under controlled experimental conditions, we performed ketamine injections in a mouse model of NT1.

### Animal Experiments

Orexin knockout mice show cataplexy and sleep fragmentation, serving as established proxies for human NT1 symptoms.^2^ Four females and 4 males were implanted for polysomnography at 12 weeks‐of‐age. Housing conditions, surgical procedures, polysomnographic recordings, vigilance state scoring, and quantification were conducted as previously described,^3^ under ethical approval (#19773).

Briefly, mice received 4 intraperitoneal injections at 6:58 PM, immediately before lights‐off: 2 injections of vehicle solution (NaCl 0.9%, 1 μl/g) on days 14 and 21 post‐surgery, and 2 injections of ketamine (10 mg/kg corresponding to one‐tenth of the dose typically used under surgical anesthesia^4^) on days 17 and 24 (Fig 1A). To elicit emotion‐triggered cataplexy (Fig 1C), mice were given 1 g of chocolate in addition to their standard diet^3^ at lights‐off. As cataplexy occurs only during the dark phase in mice, polysomnography recordings were acquired during the dark phase following each injection. Analyses focused on the first 4 h of recordings (7:00 PM to 11:00 PM). Collected data were average per mouse, across both conditions. Cataplexy characteristics (time spent in cataplexy [minutes], duration of cataplexy [seconds], number of cataplexy) and sleep parameters (for REM and non‐REM sleep: total duration [minutes], mean episode duration [minutes], and number of episodes) were compared between conditions using Wilcoxon tests with GraphPad Prism (version 10.6.1; Boston, MA, USA).

**FIGURE 1:**
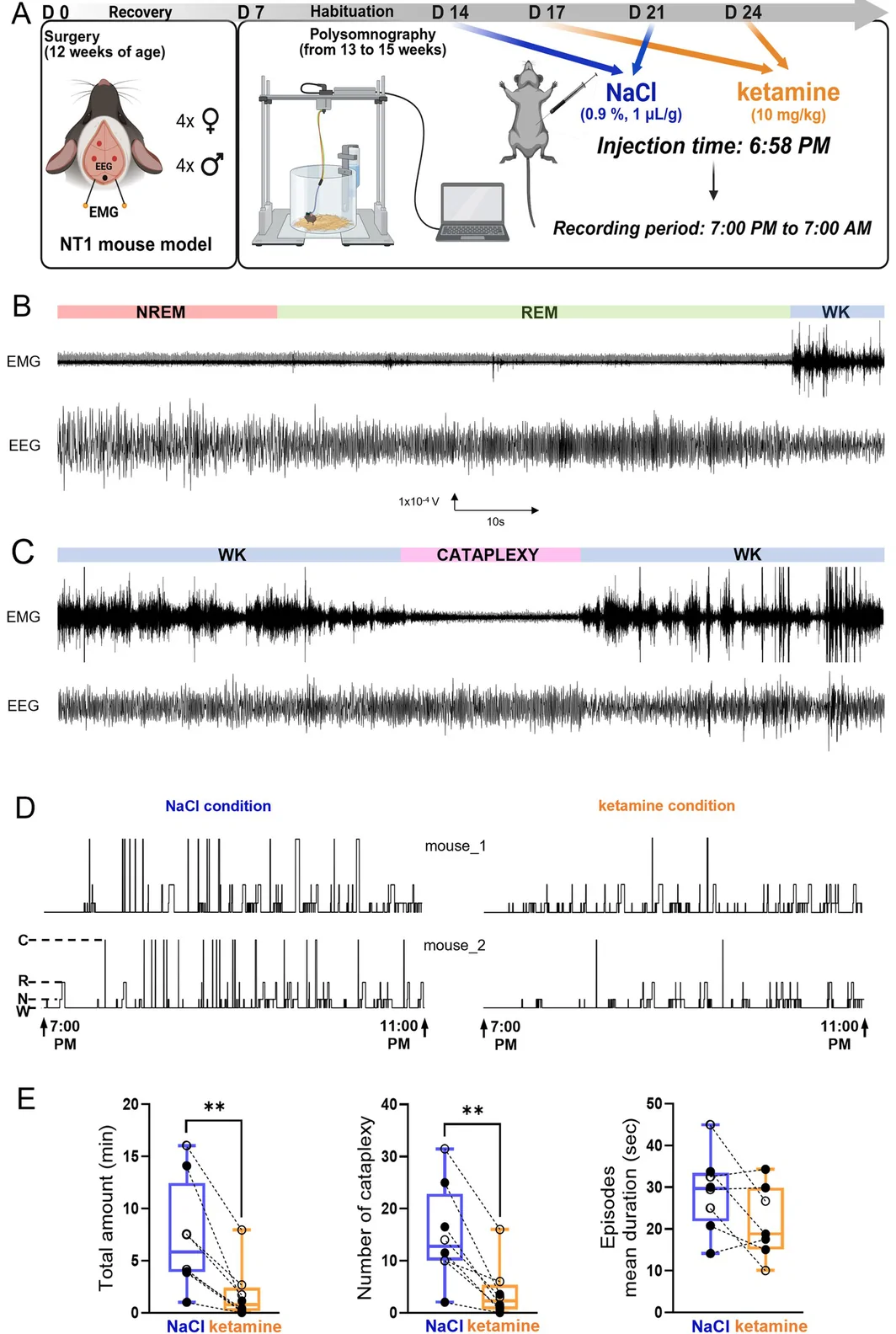
Experiments performed in narcolepsy type 1 mice and cataplexy outcomes. (A) Experimental design. Graphic design: BioRender. (B, C) Representative 100‐second electroencephalographic (EEG) and electromyographic (EMG) traces from the same animal, and the same 4‐hour recording. (B) Succession of non‐rapid eye movement (NREM), rapid eye movement (REM), and wakefulness (WK) states. (C) Cataplexy episode occurring between 2 periods of active wakefulness (WK). Identification of the 20‐second cataplexy episode was corroborated by video monitoring, which showed the animal being highly active, followed by a sudden behavioral arrest and loss of posture, and then an equally sudden resumption of active wakefulness. (D) Representative hypnograms of 2 narcolepsy type 1 mice during the analyzed period (7:00 PM to 11:00 PM) following intraperitoneal injections of NaCl or ketamine. States represented: W = wakefulness; N = non‐rapid eye movement (NREM) sleep; R = rapid eye movement (REM) sleep; C = cataplexy. (E) Box plots showing comparisons between NaCl and ketamine conditions for cataplexy amounts (=time spent in cataplexy, minutes), number of episodes and mean episode duration (sec) during the first 4‐h post injection. Data from all mice (*n* = 8) were pooled, as no sex differences were observed. Empty and filled dots represent females and males, respectively. Black dashed lines connect the paired values. **p < 0.01. Graphs and statistics were performed using Wilcoxon tests with GraphPad Prism (version 10.6.1). [Color figure can be viewed at www.annalsofneurology.org]

## Results

Representative hypnograms are shown in Fig 1D. Ketamine administration induced a profound reduction in time spent in cataplexy compared with vehicle (1.77 ± 0.9 minutes vs 7.29 ± 1.9 minutes; *p* = 0.0078; Fig 1E). This reduction was primarily explained by a marked decrease in the number of cataplexy episodes (3.94 ± 1.9 vs 15.06 ± 3.3, *p* = 0.0078), whereas mean episode duration remained unchanged (*p* = 0.2188). This decrease in cataplexy amount (−63%, *p* = 0.0156) and number of episodes (−61%, *p* = 0.0156) was also seen when comparing ketamine condition versus baseline without chocolate (1.77 ± 0.9 minutes vs 4.7 ± 0.9 minutes and 3.94 ± 1.9 vs 10.12 ± 1.9, respectively), demonstrating that the effect of ketamine is not due to a reduced consumption of chocolate. No difference in wakefulness, non‐REM sleep, or REM sleep parameters (total duration, number of episodes, and mean episode duration) was observed during this period (Fig 2).

**FIGURE 2:**
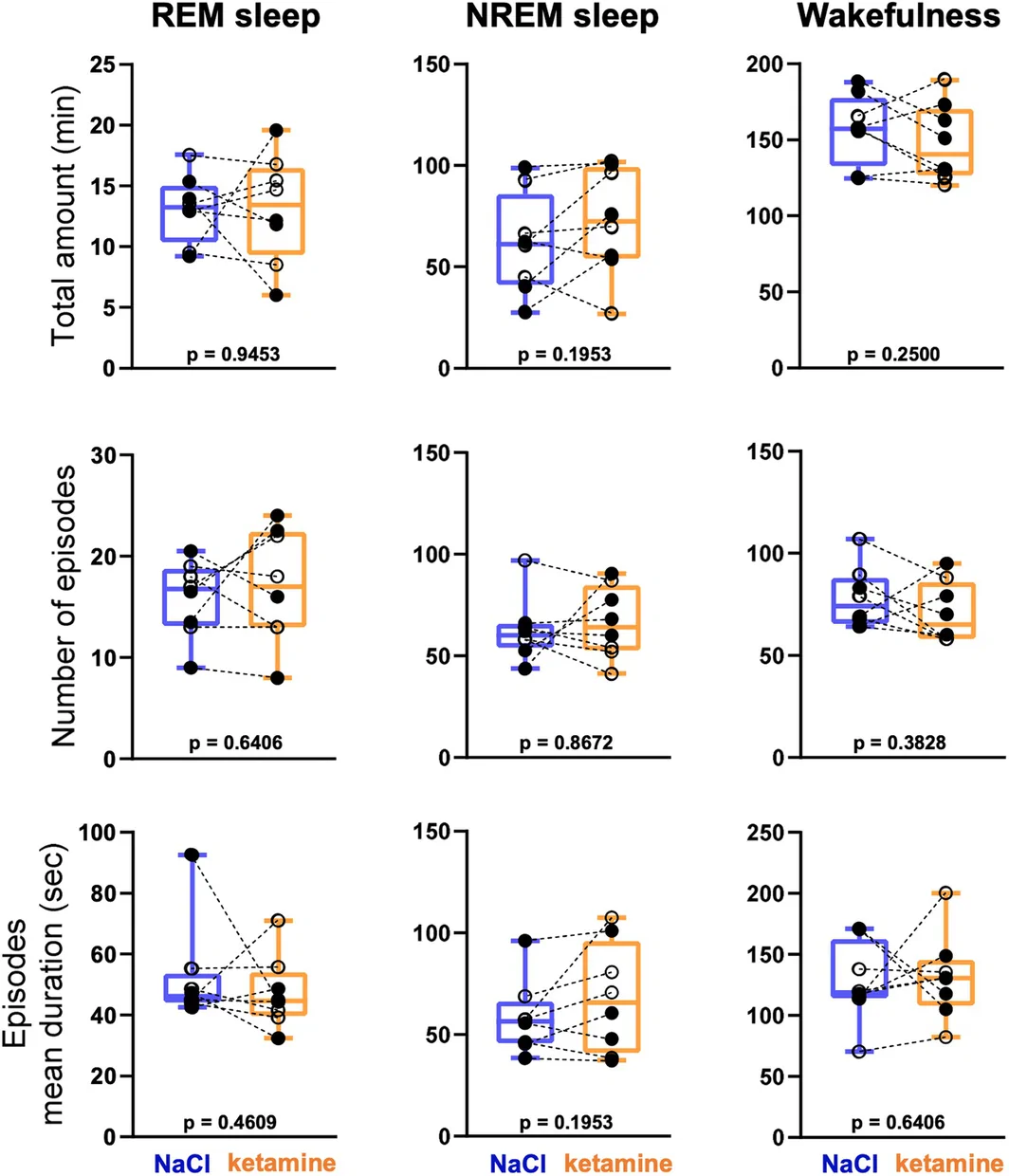
Quantification of vigilance states during the 4‐hour period following i.p. injection of NaCl or ketamine. Box plots showing comparisons between NaCl and ketamine conditions for the amounts (minutes), number of episodes, and mean episode duration (seconds) for the 3 vigilance states (rapid eye movement [REM] sleep, non‐rapid eye movement [NREM] sleep, wakefulness). Data from all mice (*n* = 8) were pooled, as no sex differences were observed. Empty and filled dots represents females and males, respectively. Black dashed lines connect the paired values. Graphs and statistics were performed using Wilcoxon tests with GraphPad Prism (version 10.6.1). [Color figure can be viewed at www.annalsofneurology.org]

## Discussion

Cataplexy manifests as a sudden and transient loss of muscle tone with preserved consciousness, typically precipitated by strong emotions. It may reflect either the intrusion of REM sleep‐related atonia into wakefulness or the emergence of a distinct hybrid vigilance state.^5^ Current models converge on dysfunctional interactions between hypothalamic orexin neurons, limbic emotion‐processing circuits, and brainstem motor‐atonia generators.^6^ Here, we found that ketamine does not significantly reduce cataplexy episodes duration in orexin‐deficient mice nor modulate REM sleep. However, ketamine strongly reduces the number of cataplexy episodes, suggesting an action on the initiation of cataplexy rather than on REM sleep circuits.

The emotional gating of cataplexy is thought to involve the medial prefrontal cortex, specifically the anterior cingulate and prelimbic cortices, which interface with reward‐ and emotion‐related networks.^7^ Among these, the nucleus accumbens—a central hub of reward and motivational processing—has been directly implicated in the initiation of positive emotion‐induced cataplexy in animal models.^8^, ^9^, ^10^ In parallel, activation of GABAergic neurons within the amygdala contributes critically by inhibiting brainstem regions that normally suppress muscle atonia.^11^ These brainstem structures are physiologically activated by orexinergic input, which is lost in NT1.^12^

Ketamine is a non‐competitive N‐methyl‐D‐aspartate receptor antagonist with anesthetic, analgesic, and antidepressant properties mediated by complex and multimodal neurobiological mechanisms. Its primary effect involves disinhibition of cortical glutamatergic transmission, leading to increased presynaptic glutamate release.^13^ These mechanisms are particularly relevant in narcolepsy, as glutamate is co‐released by orexin neurons, and glutamatergic projections extensively innervate the prefrontal cortex, thalamus, limbic structures, and hypothalamus. Beyond glutamate, ketamine modulates several neuromodulatory systems that are critically involved in cataplexy control, including noradrenergic, dopaminergic, and serotonergic pathways, and engages opioid signaling under specific conditions.^13^ Thus, ketamine may exert anticataplectic effects at multiple levels. First, it may modulate medial prefrontal cortex activity, reducing hyperactivity of the anterior cingulate cortex and enhancing top‐down inhibitory control over the amygdala, as demonstrated in stress‐related disorders, such as post‐traumatic stress disorder.^14^, ^15^ Second, by attenuating amygdala reactivity to emotional stimuli,^16^ ketamine may potentially dampen emotional triggers of cataplexy. Third, ketamine partially engages κ‐opioid receptor signaling within limbic circuits, including the nucleus accumbens and the amygdala; κ‐opioid activation is known to reduce emotional and stress‐related reactivity, opioid compounds have demonstrated anticataplectic efficacy in animal models, and some reports suggest a possible efficacy in humans with NT1.^17^, ^18^, ^19^ Finally, ketamine activates serotonergic neurons in the dorsal raphe nucleus, a pathway known to inhibit cataplexy by suppressing amygdala‐driven motor atonia.^20^, ^21^

Taken together, our observations suggest that ketamine may exert anticataplectic effects through converging actions. The case report, supported by translational experimental evidence, highlights a novel therapeutic avenue for cataplexy, warranting further clinical investigation.

## Author Contributions

F.R., S.A., L.P.D., and C.P. contributed to the conception and design of the study. F.R., A.R.M., and H.D. contributed to the acquisition and analysis of data in human. S.A., L.L., S.M., and C.P. contributed to the acquisition and analysis of data in animals. F.R., S.A., B.R., L.P.D., and C.P. contributed to drafting a significant portion of the manuscript or figures.

## Potential Conflicts of Interest

Nothing to report.

## Data Availability

The data that support the findings of this study are available from the corresponding author upon reasonable request.
